# Moderate intensity physical activity prevents increased blood glucose concentrations, fat pad deposition and cardiac action potential prolongation following diet-induced obesity in a juvenile-adolescent rat model

**DOI:** 10.1186/2052-9538-1-11

**Published:** 2014-08-20

**Authors:** Alannah van Waveren, Mitch J Duncan, Fiona R Coulson, Andrew Fenning

**Affiliations:** Central Queensland University, Institute of Health and Social Science Research, Rockhampton, Queensland 4702 Australia; School of Medicine & Public Health, Priority Research Centre in Physical Activity and Nutrition, The University of Newcastle, Newcastle, Australia; School of Medical and Applied Sciences, Central Queensland University, Rockhampton, Queensland 4702 Australia

**Keywords:** Western obese diets, Juvenile obesity, Metabolic syndrome, Physical activity, Hypertension, Oxidative stress, Inflammation, Vascular reactivity, Cardiac arrhythmia

## Abstract

**Background:**

Both obesity and a lack of physical activity have been associated with an elevated risk of cardiovascular disease (CVD). The incidence of obesity is increasing, especially in juvenile-adolescents. While there is limited research examining the chronic effects of obesity in adolescent humans and animal models of this condition, little is also known concerning how moderate physical activity might prevent or attenuate secondary cardiovascular complications induced by obesity during adolescence. We investigated the effects of diet-induced obesity (consisting of a high-fat, high-carbohydrate diet (HFHC)) on biometric indices, vascular and airway function, cardiovascular function, systemic oxidative stress and markers of inflammation in a juvenile-adolescent rodent model. Four groups were used: control (CON), physical activity (PA) treated, HFHC and HFHC + PA (n = 16 per group). HFHC feeding started at 4 weeks of age for a period of 12 weeks. Physical activity treatment was initiated (PA and HFHC + PA groups) when the animals were 8 weeks of age, for 8 weeks.

**Results:**

Physical activity in juvenile-adolescent healthy rats showed no change in comparison to the CON group in all experimental parameters except for increases in lipid peroxidation, decreases in inflammatory cytokines, improvements in vascular reactivity and decreased atrial responses to positive chronotropic agents. The HFHC animals were mildly hyperglycemic, hypertensive, displayed renal hypertrophy and showed increased retroperitoneal fat pad deposition compared to the CON group. HFHC + PA rats were also hypertensive, however showed improvements in cardiac electrophysiology, body weight, fat pad deposition and inflammatory signaling, in comparison to the HFHC fed rats and CON animals.

**Conclusion:**

In conclusion, in a juvenile-adolescent animal model of diet-induced obesity engagement in physical activity is beneficial in reducing the inflammatory effects of obesity.

## Background

In recent decades the prevalence of overweight youth and obesity in humans has increased [1, 2]. Juvenile onset obesity has been associated with adverse changes in cardiovascular risk in both animals and humans ([3–5]). Animal studies show that cardiorespiratory function and blood pressure are adversely altered following juvenile-onset obesity in comparison to older models of obesity [6–8]. In addition, following high fat feeding that induced obesity; blood pressure in the young rats became significantly elevated [6, 8]. This increase resulted in a more advanced metabolic syndrome in contrast to the older animals fed a similar diet, over a similar time period [6, 8]. Obesity has also been shown to adversely alter respiratory physiology, including a heightened demand for ventilation, diminished respiratory compliance, elevated work of breathing and respiratory muscle inefficiency [9–11].

Adipose tissue plays a key role in the secretion of many pro-inflammatory cytokines [12–14] with higher body fat deposits promoting an excess secretion of adipokines [15]. These released adipokines promote a raft of systemic effects including enhanced appetite, insulin resistance, altered bone metabolism, altered endocrine and reproductive function, decreases in pulmonary function and an increased risk of CVD [12–14, 16, 17]. Therefore, a reduction in adipose tissue generates an anti-inflammatory state, which reduces CVD risk. Reduction in adipose tissue is typically achieved by increases in physical activity, improved dietary habits or a combination of both.

Physical activity has been defined as by the World Health Organization as any bodily movement produced by skeletal muscles that requires energy expenditure [18]. Exercise, also defined by the World Health Organization, is a subcategory of physical activity defined as being planned or structured and can be repetitive [18]. Exercise is assumed to have a final objective being the improvement or maintenance of physical fitness [18]. Physical activity in daily life includes exercise and can be categorized into other activities which involve bodily movement and are done as part of playing, occupation, active transportation, household chores and sports [18].

The health benefits of regular physical activity occur via many pathways including improved regulation of metabolic irregularities, blood pressure, glucose clearance, myocardial energetics, coronary artery diameter and vasomotor tone [19, 20]. Physical activity has also been found to have anti-inflammatory effects linked to a reduction in risk of atherosclerosis and CVD [19] through decreases in the release of pro-inflammatory cytokines [21–23]. Whereas, weight-loss associated with physical activity confers a significant health benefit and has been shown to improve endothelial dysfunction and decrease systemic inflammation [24]. Studies in rodents showed that swimming for 2 hours/day, 5 days/week for 6 weeks reduced insulin resistance induced via a high fat diet [25]. Similarly, treadmill-running studies using rats, where animals completed 13 weeks of treadmill-running 5 days/week demonstrated that physical activity decreased hypertension caused by diet-induced obesity [3]. Whilst these studies have demonstrated that physical activity can reduce markers of cardiometabolic, CVD risk following diet-induced obesity in adult animal models, it is unknown how physical activity impacts CVD risk in youth.

The aims of this study were to 1) examine if diet-induced obesity causes metabolic syndrome and associated secondary CVD complications such as elevated blood pressure and blood glucose, depressed cardiovascular and respiratory function and enhanced systemic inflammation and oxidative stress in a juvenile-adolescent rat model and 2) to examine if physical activity can reduce these adverse changes.

## Methods

### Animals and animal care

All experimental procedures were approved by the CQUniversity Animal Ethics Research Committee (approval A10/11-265) and were conducted in accordance with the National Health and Medical Research Council (NHMRC) guidelines. Male Wistar rats were randomized into one of four experimental groups: 1) control (CON), 2) physical activity (PA), 3) high fat/high carbohydrate (HFHC) and 4) high fat/high carbohydrate combined with physical activity (HFHC + PA). Animals were started treatment at the juvenile age of 4 weeks [26, 27]. HFHC was initiated at 4 weeks of age for a period of 12 weeks. Physical activity was initiated (PA and HFHC + PA) when the animals reached 8 weeks of age for a period of 8 weeks. All animals were euthanized at 16 weeks of age. CON and PA animals were fed standardized rat and mouse nuts (Norco Stockfeeds; South Lismore, NSW, Australia) and were exposed to room air.

### Physical activity protocol

Groups subjected to physical activity (PA and HFHC + PA), performed 30 minutes of moderate intensity exercise per day, five days per week for 8 weeks. This daily duration and intensity is consistent with the level of physical activity considered to confer health benefits in humans [28, 29]. Studies have shown that a moderate intensity of physical activity for the rodent model is equivalent to 0.8 km/hour on a modified treadmill (AccuScan Instruments, Columbus, Ohio, USA), and is sufficient to induce physiological adaptations in cardiovascular function and biochemical parameters [30, 31]. HFHC and CON groups were not subjected to physical activity, however where exposed to the same condition inside the treadmill unit.

### HFHC diet-induced obesity model

A modified HFHC diet [32, 33] was prepared by Specialty Feeds (Glen Forrest, WA, Australia) and consisted of 45.3% digestible energy from lipids and 34.3% from carbohydrates. The HFHC diet pellets contained casein (233 g/kg), fructose (175 g/kg), lard (207 g/kg), soya bean oil (29 g/kg), cellulose (58 g/kg), wheat starch (118 g/kg), dextrinized starch (117 g/kg), DL methionine (3.5 g/kg), calcium carbonate (6.4 g.kg), sodium chloride (2.6 g/kg), AIN93 trace mineral (1.6 g/kg), potassium citrate (19.2 g/kg), di-calcium phosphate (15.1 g/kg), potassium sulphate (1.6 g/kg), choline chloride 75% (1.3 g/kg) and AIN93 vitamins (12 g). Digestible energy was calculated at 19.5 MJ/kg. The CON and PA did not receive the modified HFHC diet but standard rat chow. kJ consumption was calculated by weighing food and water intake daily. The consumption was then averaged based on the number of rats present in each cage and multiplied by kJ content in the food or water that was eaten.

### Body mass, heart rate and systolic blood pressure

Systolic blood pressure and heart rate readings were taken 0, 4, 8 and 12 weeks. Systolic blood pressure and heart rate were measured via tail cuff plethysmography as outlined previously [34]. Body mass was measured weekly for all groups.

### Terminal assessments

At completion of the 12-week treatment regime, all rats were euthanized using a 0.2 ml/kg i.p. injection of sodium pentobarbitone (375 mg/ml). Following euthanasia, the abdominal and chest cavities were opened and a 5 mL blood sample was collected from the abdominal vena cava. Internal organs including the heart, kidneys, liver, spleen and fat pads were removed and weighed.

### Glucose, 4-HNE, NO, IL-6 and IL-1B

Blood glucose concentrations for each group were measured immediately following euthanasia using a blood glucose monitor (Medisense, Abbott Laboratories) and reported in mmol/L. All other biochemical measurements were performed using serum prepared from whole blood collected in a serum separator tube and allowed to clot before being centrifuged. Samples were then centrifuged for ten minutes at 14000 rpm and the supernatant removed and stored at −80°C to prevent sample degradation. To assess 4-hydroxynonenal (4-HNE) levels for each animal, serum samples were used in conjunction with a Cell Biolabs’ Oxiselect™ HNE adduct ELISA kit (Catalog Number STA-338). Nitric Oxide (NO) levels were assessed using a NO (total) Detection Kit (Catalog Number ADI-917-020). Assessments of serum interleukin-1β (IL-1β) concentrations were made through the use of a R&D Systems Quantikine Rat IL-1β/IL-1 F2 Immunoassay (Catalog Number RLB00) with serum interleukin-6 (IL-6) concentrations determined through the use of R&D Systems Quantikine Rat IL-6 Immunoassay (Catalog Number R6000B).

### Vascular reactivity in isolated tissues

Thoracic aortic rings isolated from each rat were cleared of any fat and connective tissue before being suspended in 25 mL organ baths stabilized at 37°C with a continuously supplied with carbogen (5% CO_2_ and 95% O_2_) gas bubbled through tyrodes solution (NaCl 136.9, KCl 5.4, MgCl_2_ 1.05, NaH_2_PO_4_ 0.42, NaHCO_3_ 22.6, CaCl_2_ 1.8, glucose 5.5, ascorbic acid 0.28, EDTA 0.1 all in mM). Each segment of thoracic aorta had a pre-set resting tension of 10 mN and following a thirty minute equilibration period, cumulative concentration response curves (CRC) to noradrenaline (NA), acetylcholine (ACh) (NA pre-contraction) and sodium nitroprusside (Nano) (NA pre-contraction) were established with any fluctuation to the preset tension recorded using (Grass FT03) transducers connected to Chart software (21). Vascular reactivity in isolated pulmonary arteries was assessed using a 4-bath wire-myograph system (Danish Myograph Technologies, Denmark). Pulmonary arteries were dissected from the base of the heart and threaded with 40 μm stainless steel wire while bathed in cold Tyrodes buffer and gassed with carbogen before being transferred to the myograph chambers at 37°C. Once the pulmonary tissues had equilibrated for 20 minutes they were normalized using inbuilt normalization protocols of LabChart Pro 7 software (ADInstruments). Following successful normalization, tissues were rested at pre-load tension 10 mN, for 20 minutes before being contracted with 10 mM KCL and relaxed with a 1e-5 M concentration of ACh. The pulmonary arteries were washed regularly for 30 minutes with fresh buffer solution before the commencement of the same CRC’s to the thoracic aortic rings.

### Assessment of cardiac electrophysiological changes

The papillary muscle was excised from the left ventricle and a stainless steel hook inserted through the superior end. The papillary muscle was then placed between two platinum electrodes in a 1.0 mL experimental chamber filled with Tyrodes physiological salt solution (37°C; aerated with carbogen) and fixed into position with a stainless steel pin (21). The papillary muscle was then slowly stretched to a maximum pre-load (5 mN) and then was then stimulated using a Grass SD-9 stimulator and contractions were induced at 1 Hz, with a pulse width of 0.5 msec and stimulus strength 20% above threshold. After a five-minute equilibration period, the papillary muscle was then impaled by a glass electrode filled with potassium chloride 1 M (filamented borosilicate glass, outer diameter 1.5 mm, tip resistance of 5-15 mΩ when filled with 3 M KCL), using a silver/silver chloride reference electrode. The electrical activity, which was recorded in mV, of a cell was recorded following a further 25 minute equilibration period with a Cyto 721 electrometer was connected to an ADInstruments (Chart 7) recording system.

### Assessment of pacemaker changes

The right atria were isolated from each rat and were cleared of any fat and connective tissue was removed before being suspended in 25 mL organ baths stabilized at 37°C, filled with Tyrodes physiological salt solution (aerated with carbogen). Each atrium had a thirty-minute equilibration period, then cumulative concentration response curves (CRC) to isoprenaline (ISO) and calcium chloride (CaCl) were added with any fluctuation to mediate rate response of recorded using (Grass FT03) transducers connected to Chart software.

### Assessment of airways function in isolated sections of trachea and bronchioles

Trachea rings isolated from each rat were cleared of any fat and connective tissue before being suspended in 25 mL organ baths [35] stabilized at 37°C supplied with carbogen gas bubbled through modified KHB (NaCl 119.1, KCl 4.75, MgSO_4_ 1.19, KH_2_PO_4_ 1.19, NaHCO_3_ 25.0, glucose 11.0 and CaCl_2_ 2.16 all in mM) with added propranolol to replicate biological parameters. Each segment of trachea had a preset resting tension of 1 g and following a 30-minute equilibration period, CRC to carbachol (CAR), 5-hydroxytryptamide (5-HT) and isoprenaline (ISO) were completed. Pulmonary responses in isolated bronchioles were assessed via a 4-bath wire-myograph system (Danish Myograph Technologies, Denmark). Second generation bronchioles were dissected from the lungs and threaded with 40 μm stainless steel wire while bathed in cold (10°C) Krebs-Henseleit buffer and gassed with carbogen before being transferred to the myograph chambers at 37°C. Equilibration and normalization procedures were kept consistent with the pulmonary artery preparations. CRC were performed using 5-HT, ACh and ISO (ACh pre-contraction).

### Drugs and chemicals

All drugs used in this study (NA, ACh, CAR, 5-HT, ISO and NaNO) were purchased from the Sigma Chemical Company, St Louis MO, USA. Serial dilutions of the drugs were produced using distilled water.

### Statistical analysis

Data is expressed as mean ± standard error mean (SEM). Statistical analysis was performed using two-way analysis of variance (ANOVA) and students t-test where appropriate. Results were considered significant when P < 0.05 with analysis carried out using Graphpad Prism v5 (GraphPad Software La Jolla, CA 92037 USA).

## Results

### Responses following PA

**Figure 1:**
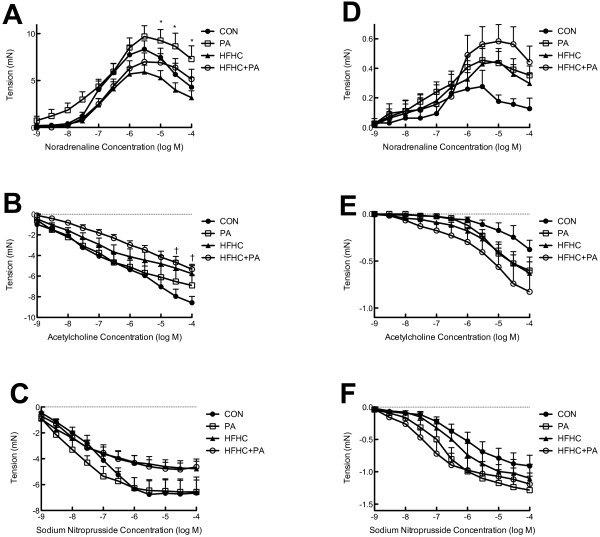
**Vascular functional changes. A**, Noradrenaline mediated contraction of the thoracic aorta. **B**, Endothelium-dependent relaxation by acetylcholine of noradrenaline pre-contracted thoracic aorta. **C**, Endothelium-independent relaxation by sodium nitroprusside of noradrenaline pre-contracted thoracic aorta. **D**, Noradrenaline mediated contraction of the pulmonary artery. **E**, Endothelium-dependent relaxation by acetylcholine of noradrenaline pre-contracted pulmonary artery. **F**, Endothelium-independent relaxation by sodium nitroprusside of noradrenaline pre-contracted pulmonary artery. Data expressed as Mean ± SEM; n = 16 for all groups. *P < 0.05 PA vs HFHC, ^†^P < 0.05 CON vs HFHC + PA.

**Figure 2:**
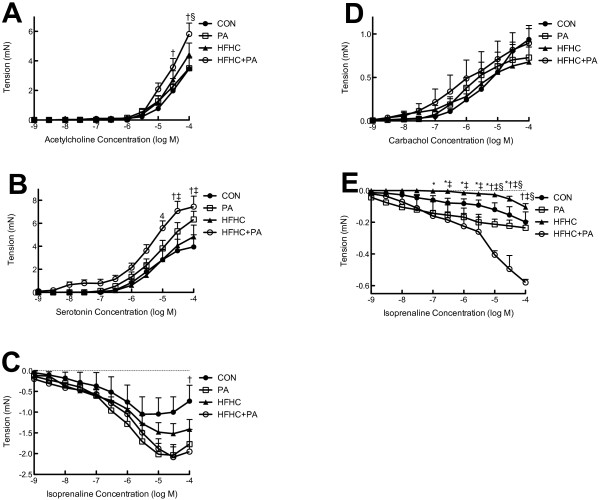
**Small and large airway functional changes. A**, Acetylcholine mediated contraction of the bronchioles. **B**, Serotonin mediated contraction of the bronchioles. **C**, Isoprenaline mediated relaxation acetylcholine pre-contracted bronchioles. **D**, Carbachol mediated contraction of the trachea of control. **E**, Isoprenaline mediated relaxation on carbachol pre-contracted trachea of control. Data expressed as Mean ± SEM; n = 16 for all groups. *P < 0.05 PA vs HFHC, ^†^P < 0.05 CON vs HFHC + PA, ^‡^P < 0.05 HFHC vs HFHC + PA, ^§^P < 0.05 PA vs HFHC + PA.

No significant changes were observed in body mass in comparison to age- matched controls. Similar results were found in the fat pads and organ masses (Table 1). PA caused a significant increase (P < 0.05) in lipid peroxidation, however a significant decrease (P < 0.05) in circulating cytokine (IL-6 and IL-1β) was also observed (Table 1).Physical activity resulted in an enhanced aortic response to noradrenaline however the relaxation responses to ACh and NaN were unchanged (Figure 1A). Whilst not significant there was a trend that physical activity enhanced both the relaxation and contraction responses in bronchiole tissues (Figure 2).

**Table 1:** **Systemic parameters in the four intervention groups**

	CON	PA	HFHC	HFHC + PA
Body weight (g)	444.8 ± 7.6	465.5 ± 8.9	441.1 ± 9.0	422.9 ± 8.0^†^
Retroperitoneal (g)	7.8 ± 1.1	7.9 ± 0.6	12.6 ± 1.2*^†^	9.0 ± 0.7
Subcutaneous (g)	8.3 ± 1.0	8.5 ± 0.7	10.1 ± 0.6	8.9 ± 1.3
Epididymal (g)	8.5 ± 1.0	6.2 ± 0.3	7.6 ± 0.7	7.8 ± 0.5
Mesenteric (g)	7.6 ± 0.9	8.5 ± 0.7	8.2 ± 0.5	7.4 ± 0.4
LV and septum (mg/g of bw)	2.2 ± 0.08	2.0 ± 0.08	2.1 ± 0.06	2.2 ± 0.08
RV (mg/g of bw)	0.5 ± 0.05	0.4 ± 0.03	0.5 ± 0.05	0.5 ± 0.02
Liver (mg/g of bw)	36.9 ± 0.7	36.8 ± 0.6	36.6 ± 0.9	37.5 ± 3.0
Spleen (mg/g of bw)	2.8 ± 0.1	2.7 ± 0.1	2.7 ± 0.1	2.9 ± 0.1
Kidneys (mg/g of bw)	7.6 ± 0.2	7.1 ± 0.1	8.2 ± 0.2*^†^	8.5 ± 0.2*^†^
4-HNE (mmol/L)	0.4 ± 0.2	10.4 ± 0.9*^‡^	3.6 ± 0.8*^§^	12.2 ± 1.7*^‡^
IL-6 (pg/mL)	770.0 ± 91.9	194.3 ± 31.5*^‡^	620.0 ± 91.7	417.1 ± 35.6*
IL-1β (pg/mL)	105.4 ± 13.6	49.4 ± 8.8*	67.2 ± 12.0	52.9 ± 3.9*
Nitrate/Nitrite (μmol/L)	22.6 ± 1.2	33.6 ± 2.7	68.8 ± 5.2*^†§^	34.5 ± 8.5
Glucose (mmol/L)	12.0 ± 1.1	11.9 ± 1.0	15.1 ± 0.6*^†^	12.4 ± 1.0

Data expressed as Mean ± SEM; n = 16 for all groups. CON, control; PA, physical activity treated; HFHC, high-fat, high-carbohydrate fed; HFHC + PA, high-fat, high-carbohydrate fed and physical activity treated. *P < 0.05 vs CON, ^†^P < 0.05 vs PA, ^‡^P < 0.05 vs HFHC, ^§^P < 0.05 vs HFHC + PA.

**Figure 3:**
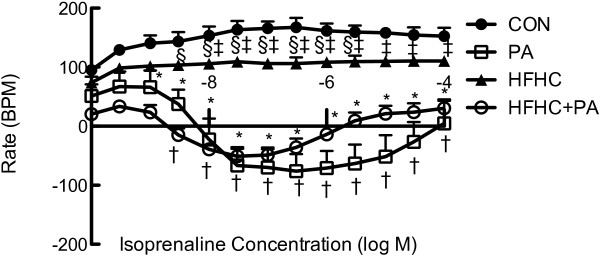
**Isoprenaline mediated rate response of the right atria.** Data expressed as Mean ± SEM; n = 16 for all groups. *P < 0.05 CON vs HFHC + PA, ^†^P < 0.05 CON vs PA, ^‡^P < 0.05 PA vs HFHC, ^§^P < 0.05 HFHC vs HFHC + PA.

Physical activity reduced ISO stimulated increases in heart rate from the right atria (Figure 3A) but did not alter any cardiac electrophysiological parameters (Table 2).

**Table 2:** **Resting membrane potential (RMP), action potential amplitude (AMP), action potential duration at 20% (ADP20), action potential duration at 50% (ADP50) and action potential duration at 90% (ADP90) of repolarization**

	CON	PA	HFHC	HFHC + PA
RMP	−69.5 ± 1.7	−64.1 ± 1.7	−67.1 ± 1.2	−62.4 ± 1.8*
APA	66.2 ± 2.8	65.6 ± 2.7	68.5 ± 2.1	64.3 ± 2.8
APD20	23.6 ± 0.5	24.5 ± 0.3	25.6 ± 0.7	23.9 ± 0.6
APD50	32.2 ± 1.4	34.0 ± 1.6	38.6 ± 1.4*	33.2 ± 1.1^‡^
APD90	70.6 ± 5.8	77.2 ± 0.4	91.9 ± 3.9*^†^	77.9 ± 4.2^‡^

Data expressed as Mean ± SEM; n = 16 for all groups. *P < 0.05 vs CON, ^†^P < 0.05 vs PA, ^‡^P < 0.05 vs HFHC.

### Responses following HFHC feeding

**Figure 4:**
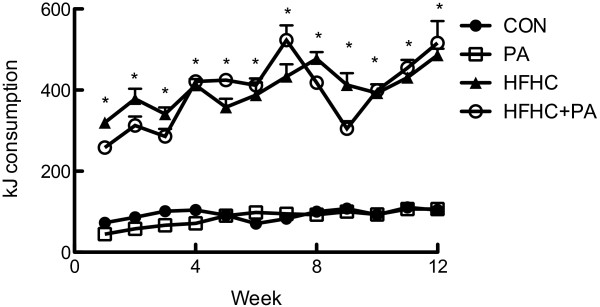
**kJ intake.** Data expressed as Mean ± SEM; n = 16 for all groups. *P < 0.05 CON vs HFHC, HFHC + PA and PA vs HFHC, HFHC + PA.

**Figure 5:**
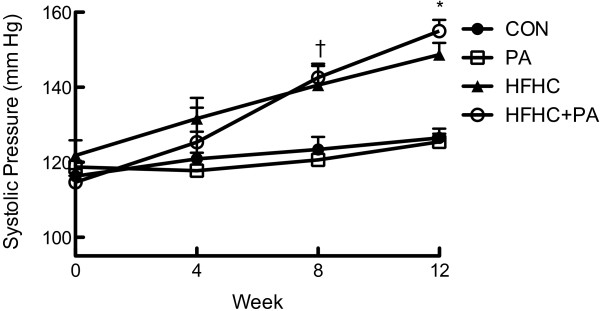
**Systolic blood pressure.** Data expressed as Mean ± SEM; n = 16 for all groups. *P < 0.05 CON vs HFHC and HFHC + PA, ^†^P < 0.05 PA vs HFHC.

HFHC feeding did not increase body mass in the juvenile-adolescent rats, however the diet did significantly increase retroperitoneal fat pad mass compared to the CON animals (Table 1). HFHC feeding also increased kidney mass but no other organ hypertrophy was observed (Table 1).There was a significantly increased kJ intake in the HFHC groups compared to both standard chow fed animals (CON and PA) (Figure 4). Hypertension was induced by the HFHC diet with a significant increase of 9% increase after 4 weeks, 14% after 8 weeks and 20% (P < 0.05) in systolic blood pressure after 12 weeks of feeding (Figure 5).

HFHC also showed a significant increase (P < 0.05) in 4-HNE values with no change in IL-6 and IL-1β levels (Table 1). The animals in this group were characterized by having significantly higher blood glucose values when compared to the CON experiments (Table 1).

The action potential duration in the papillary muscles were found to be significantly (P < 0.05) prolonged in the HFHC group by 20% at 50% of repolarization and 30% at 90% of repolarization compared to the CON animals (Table 2).HFHC feeding did not significantly alter (P > 0.05) aortic (Figure 1) or respiratory (Figure 2) tissue responses but did show a significantly reduced right atrial response to ISO (Figure 3A).

### Responses following HFHC feeding and PA

HFHC + PA rats showed a significant decrease (P < 0.05) in body mass compared to HFHC with a normalized retroperitoneal and subcutaneous fat pad mass (Table 1). However, physical activity was not able to normalize renal hypertrophy following HFHC feeding (HFHC + PA) (Table 1).There was a significantly increased kJ intake in the HFHC + PA animals compared to both standard chow fed groups (CON and PA) (Figure 4). Systolic blood pressure was significantly increased by 23% in the HFHC + PA group in comparison to the CON group (Figure 5).

The HFHC + PA group showed significantly increased levels of 4-HNE, IL-6 and IL-1β levels compared to CON (Table 1). There was no significant difference in blood glucose levels between HFHC and HFHC + PA rats or between HFHC + PA and CON (Table 1).

Physical activity was able to normalize the prolonged APD at 50 and 90% of repolarization observed in the HFHC fed animals (Table 2). Physical activity in the HFHC fed animals (HFHC + PA) decreased the atrial responses to the positive chronotropic agent ISO to a similar degree in PA group (Figure 3).The HFHC + PA group showed exaggerated reaction to both relaxation and contraction response curves in the thoracic aorta and pulmonary arteries (Figure 2).

## Discussion and conclusions

Moderate physical activity was able to prevent some of the maladaptive changes associated with chronic HFHC feeding in young rats, including, however it did not prevent an elevation in blood pressure. Non-significant trends in the data showed some improvement in vascular contractile responses to noradrenaline. Electrophysiological function at 50% and 90% of the action potential duration was significantly increased by HFHC feeding, With the HFHC + PA group values not significantly different to the CON tissues. Moderate intensity physical activity in young healthy rats (PA group) produced the typically expected changes of a reduction in systemic inflammation and maintenance of body weight and systolic blood pressure similar to CON values. In contrast, juvenile-adolescent normotensive rats fed a HFHC diet were mildly hyperglycemic, hypertensive and showed increased renal hypertrophy. Hearts from HFHC fed animals showed significantly prolonged action potentials with systemic increased lipid peroxidation as a common mediator of the metabolic syndrome. The presentation and degree of damage was not as large as reported in other studies, which may be due to the younger age of the animal model and the duration of HFHC feeding [32, 36, 37].

In this study, there was no significant gain in body mass in the HFHC group, despite an excess in kilojoules consumed compared to the CON animals. The amount of weight gain in the HFHC group observed in the current study is similar to a study that used a high fat diet over 12 weeks [38]. However, longer feeding periods (16 weeks) elucidated a larger weight gain response in HFHC fed animals [32]. Although not measured in the current study the weight gain may have been in relation to leptin resistance and appetite regulation. Leptin is an adipocyte-derived peptide, the production of which is increased in patients with obesity [39]. A recent study examined a model of diet-induced obesity and the response to leptin in several stages [36]. In the middle-stage (about 8 weeks), food intake reduced when the animal had an increase in leptin production and still retained central leptin sensitivity [36]. The animals seemed to control the rate of excess fat gain by significantly reducing food intake, however, despite the hypophagia, excess fat still accumulated at a reduced rate with apparent gains in energetic efficiency at least partially preventing this regulatory attempt [36]. However, in the later stages (4 months of high-fat feeding), the energy intake of the high-fat fed mice increased by 14.6% over the control fed animals, accompanied by a reduction of central leptin sensitivity [36]. Clearly, the increase in energy consumption of the HFHC diet in this study promoted changes in adipose deposition causing classical indicators of the metabolic syndrome, increased blood glucose and blood pressure, without excessive weight gain in these younger animals.

Although overall body mass was not significantly increased in the HFHC animals, there was a significant increase in retroperitoneal fat pad weight in the HFHC fed animals. This abdominal fat, has been shown to have a direct correlation with insulin resistance, and may be a better indicator of obesity related complications, than overall body mass [25, 40–44]. HFHC has shown to cause adverse cardiovascular changes in rodent models as well as in humans [32, 37, 45]. HFHC feeding in humans, rats and pig models has been shown to cause adverse cardiovascular changes including coronary endothelial dysfunction, vascular oxidative stress, hypertension and cardiomyocyte hypertrophy [3]. Although overall body mass was not decreased in the HFHC + PA animals retroperitoneal and abdominal fat mass was reduced which may offer cardiometabolic benefits despite the lack of overall reduction in body mass. Although the program of physical activity was unable to significantly reduce overall body mass, retroperitoneal and abdominal, fat mass was reduced and clearly conferred cardiometabolic benefits [46, 47]. It seems that overall body weight reduction may not be the most reliable indicator of the beneficial effects of physical activity [25].

Kidney hypertrophy was observed in the HFHC group, with no other significant changes in other organ masses. In this study, physical activity, was unable to reduce the increase in mass observed in kidney weight, caused by the HFHC diet. With hypertension thought to be a leading cause of renal disease, it is not surprising that many studies have tried to find the mechanism behind this effect. Studies showing that obesity-induced hypertension in dogs is associated with a shift of renal pressure natriuresis [48] and that fructose-induced metabolic syndrome is also associated with renal disturbances characterized by renal hypertrophy [49, 50], arteriolopathy, glomerular hypertension, and cortical vasoconstriction [49]. The increase in kidney mass may potentially be due to the mild hypertension observed in the HFHC fed group.

Hypertension is one of the hallmarks of the metabolic syndrome and is induced by fructose and HFHC feeding [32, 51]. The animal model of diet-induced obesity elicits an increase in systolic blood pressure in as little as four weeks [52, 53]. In the present study, a significant increase in systolic BP was observed following 8 weeks of the HFHC diet and continued to increase until 12 weeks, similar to other studies [3, 52, 53]. One of the benefits of physical activity is a reduction in blood pressure [54, 55], which was not observed in either of the physical activity groups in this study. This result is in disagreement with other findings [3]. One study showed that 13 weeks of physical activity caused a significant decrease in BP following diet-induced obesity in rats [3]. The length of physical activity treatment (8 weeks) in the current study may not have been long enough to attenuate the change caused by the HFHC diet. The speed and pace used in the current study in normal rats does induce improved left ventricular functional performance over 6–12 weeks period [30]. A significant decrease was not seen in systolic BP, potentially due to it not yet being stabilized, alternatively BP may have stabilized at a level that was not significantly lower. The findings in this study are supported by the isolated vessel studies, which showed no change in aortic dilation responses after physical activity. Other studies showing that cardiovascular damage and steady-state hypertension by diet-induced obesity was achieved only after the 16 weeks of feeding [32, 53].

HFHC feeding showed a trend in decreasing inflammatory cytokine levels both in agreement [40, 44] and contrast to previous studies [21, 32, 37, 42]. These contrasting findings may be related to systemic inflammation as an expression of advanced obesity and insulin resistance which may not be present in animals treated for 12 weeks [44]. However physical activity significantly decreased IL-6 and IL-1 β concentrations compared to the CON and HFHC fed groups. Animal studies have shown that cytokine expression is decreased in a physical activity model, and it was hypothesized that this may occur due to increased utilization of circulating fatty acids [42]. In addition, physical activity can reverse the increased levels of pro-inflammatory cytokine expression correlated to increased body mass, despite continued consumption of a high fat diet [42]. These changes persisted even though the high fat diet/physical activity mice had no significant decrease in body weight compared to the control group [42]. A recent study in a physical activity treated obese rat model found there was reduction in cardiomyocytes with inflammatory infiltrate in comparison to inactive obese rat model [56].

Oxidative stress originates in the mitochondria from reactive oxygen and reactive nitrogen species and has been linked to most of the key steps in the pathophysiology of CVD [57]. Our results showed a significant increase in this measure in the HFHC animals compared to the CON group. Interestingly, it was also to be significantly increased in our PA group and HFHC + PA group compared to our CON and HFHC group. While the HFHC diet increased lipid peroxidation, physical activity increased lipid peroxidation even further. Physical activity has been shown to induce oxidative stress partially through increased mitochondrial turnover, however with this is an adaptation or up regulation of oxidant defenses [58, 59]. This increase in oxidative stress seen is only an issue when it is in response to other maladaptive processes like diet-induced obesity [25, 60].

The potential for cardiac arrhythmia development was increased in the young HFHC fed rats as demonstrated by a significant prolongation of the cardiac action potential. It has been found that in both obese and control rats that the four major ionic membrane currents responsible for controlling action potential duration are similar [61]. This suggests that prolonged action potential duration seen in HFHC animals was due to a different mechanism. One hypothesized theory is the absence of leptin in these animals on the ventricular sodium calcium exchange current. It has been shown that leptin receptor expression is down regulated in the hearts of obese animals compared to normal fed control animals [61]. Papillary muscles from the high fat diet fed rat hearts showed higher basal and maximum forces but a decreased recovery after a higher workload [62]. The underlying mechanism which links diet-induced obesity to the progression of mild cardiac hypertrophy is unclear [62]. It is hypothesized that obesity promotes pathological cardiac remodeling with left ventricular systolic dysfunction and an increase in myocardial stiffness, which, in turn, is probably related to afterload elevation and cardiac fibrosis [63]. Although physical activity was not able to completely reduce the prolonged action potential duration at both 50% and 90% following chronic HFHC feeding, it was able to significantly attenuate this increase. Changes seen in blood glucose, fat mass and inflammatory signaling were potential factors for this improvement but also improved calcium handling leading to increased cardiomyocyte contractility [30].

HFHC feeding was shown to cause a decreased response of the right atria to adrenergic stimulation. These changes have been hypothesized to be driven by ionic current changes [61]. Typically, expected findings occurred whereby exercise caused a decrease in heart rate response due to reduction in β-adrenergic receptors in the right atrium [64]. This also occurred during the HFHC + PA group and was a healthy adaptation.

In conclusion, HFHC feeding in young rats induced a mild metabolic syndrome characterized by elevated BP and blood glucose, along with kidney hypertrophy, adipocytokine release, oxidative stress and cardiac action potential prolongation. Changes in vascular and respiratory tissues responses were minimal and linked to the juvenile-adolescent age of the experimental model. Physical activity in the young HFHC fed animals induced improvements in CVD risk by reducing components of the metabolic syndrome including a reduction in lipid peroxidation and cardiac action potential duration, which was believed to be mediated by a reduction in systemic inflammation.
